# Genotype Distribution Characteristics and Short-Term Follow-Up of HPV Infection Among Male Outpatients in Wenzhou, China

**DOI:** 10.3390/idr18050116

**Published:** 2026-10-08

**Authors:** Xianning Dai, Chenhui Zhang, Xi Xu, Kai Xu, Zitong Qian, Xi Chen

**Affiliations:** 1 Department of Clinical Laboratory, Wenzhou People’s Hospital, Wenzhou 325041, China; dxn665803@163.com (X.D.); 15868719168@163.com (X.X.); xukai975011@163.com (K.X.); 2 Department of Reproductive Medicine, Wenzhou Maternal and Child Health Hospital, Wenzhou 325000, China; zeris0621@163.com; 3 Department of Disease Control and Prevention, Wenzhou Lucheng Center for Disease Control and Prevention, Wenzhou 325007, China; q2012067@163.com

**Keywords:** human papillomavirus, male, genotype, co-infection, follow-up, epidemiological characteristics

## Abstract

Objective: The aim of this study was to investigate the genotype distribution, co-infection patterns, and disease-specific characteristics of human papillomavirus (HPV) infection among male outpatients in Wenzhou, China. Methods: A total of 1934 male outpatients who underwent HPV genotyping at Wenzhou People’s Hospital and Wenzhou Maternal and Child Health Hospital between April 2023 and March 2026 were enrolled. Baseline infection status, clinical manifestations, genotype co-infection patterns, and six-month follow-up outcomes were analyzed. Results: The overall HPV positivity rate was 39.14%. HPV52 was the most prevalent high-risk genotype (13.61%, 95% CI: 11.12–16.43%), most frequently co-detected with HPV51 and HPV58, whereas HPV6 dominated low-risk genotypes (40.16%, 95% CI: 36.69–43.66%). HPV positivity differed significantly across disease groups (*p* < 0.001), with the highest rate observed in mucocutaneous lesions (58.07%, 95% CI: 53.89–62.18%). Single-genotype infection predominated (73.71%, 95% CI: 70.58–76.85%). Of 757 HPV-positive patients, 403 (53.24%) completed six-month follow-up, and their rates of complete clearance, isolated persistence, incident-only genotype, and superinfection were 52.61%, 28.04%, 13.40%, and 5.96%, respectively. Conclusions: These findings underscore the need for male-specific HPV screening strategies, targeted health education for high-risk populations, and age-stratified individualized follow-up protocols. Such regional epidemiological evidence may help optimize local prevention, intervention, and clinical management of HPV-associated disorders in men.

## 1. Background

Human papillomavirus (HPV) is a circular double-stranded DNA virus that infects human genitourinary epithelial cells. Persistent infection with high-risk HPV genotypes triggers epithelial hyperplasia, dysplasia and malignant transformation, severely impairing reproductive health [1]. Although HPV epidemiology and pathogenic characteristics have been well established in females, evidence regarding infection patterns, genotype distribution and clinical progression in males remains limited [2]. As key asymptomatic reservoirs and transmission vectors, males frequently harbor latent HPV infections that cause recurrent cross-transmission between sexual partners, compromising population-level cervical cancer prevention [3,4]. Male chronic HPV infection is also closely associated with genital warts, genitourinary inflammation, infertility and anogenital malignancies [5]. Since HPV preferentially infects basal keratinocytes and presents a cryptic life cycle without viremia or extensive cellular lysis, it restricts inflammatory signaling and suppresses innate immune activation, which therefore partly explains the high prevalence of asymptomatic carriage and latent infection in males. Notably, unlike standardized female HPV screening programs, routine physical examinations in China do not include HPV testing for males, resulting in insufficient and unrepresentative infection data from health-check populations. Moreover, male HPV infection can involve multiple sites of the genitourinary tract with diverse clinical presentations; accordingly, specimen collection should be symptom-directed and may include urethral discharge, urine, semen, and genital exudate. Reliance on a single fixed specimen type may miss infections at other sites. Therefore, clinical population-based research employing symptom-directed sampling with different specimen types is essential to accurately characterize male HPV epidemiological profiles and fill regional data gaps.

Current domestic and international studies on male HPV infection have several notable limitations [2,6]. Most previous investigations focused on specific high-risk subgroups or single-disease populations and lacked systematic baseline data from general male outpatients, failing to reflect real-world infection features. Existing studies primarily focus on single-genotype infections while neglecting prevalent multiple-genotype co-infections and their potential synergistic pathogenic effects [7,8]. Significant regional heterogeneity in HPV positivity and genotype spectra further reduces the generalizability of external findings owing to variations in lifestyle, health awareness and health-seeking behaviors [9]. In addition, few studies have performed standardized short-term follow-up, so the natural course of male HPV infection, including viral persistence and clearance, remains poorly defined. Uniform criteria for male high-risk population screening and risk assessment are also lacking, leading to missed latent infections, sustained viral transmission and insufficiently targeted interventions [10,11]. These deficiencies hinder precision HPV prevention and impede the development of gender-balanced reproductive health strategies.

To address these gaps, this study enrolled male outpatients from urology, andrology and reproductive medicine departments, rather than low-risk health-check or community-based healthy males. These outpatients usually presented with genitourinary symptoms or a history of high-risk exposure, representing the core population for HPV infection and transmission in real clinical settings. We comprehensively analyzed baseline infection status, clinical manifestations, genotype co-infection patterns, and six-month follow-up outcomes stratified by age and initial infection status. Based on real-world multi-department clinical data from two hospitals in Wenzhou, we clarified the population distribution and dynamic progression of male HPV infection. The findings supplement local male HPV epidemiological data, optimize risk stratification for susceptible populations, and provide practical evidence for developing targeted, gender-equitable HPV prevention strategies in this region.

## 2. Materials and Methods

### 2.1. Study Population

A retrospective analysis was conducted on 1934 male outpatients from the Department of Andrology, Department of Urology, and Department of Dermatology at Wenzhou People’s Hospital and Wenzhou Maternal and Child Health Hospital between April 2023 and March 2026. All enrolled subjects received standardized follow-up within six months after initial HPV genotyping. Repeat HPV genotyping was performed at any time during this 6-month follow-up period; participants without post-baseline HPV genotyping results were excluded from longitudinal outcome analysis. Baseline demographic information, including age and marital status, was collected. Baseline laboratory data of concurrent sexually transmitted infections (STIs) were also retrieved, including human immunodeficiency virus (HIV), *Neisseria gonorrhoeae (N. gonorrhoeae)*, *Treponema pallidum (T. pallidum)*, *Ureaplasma urealyticum (U. urealyticum)*, *Chlamydia trachomatis (C. trachomatis)*, *Mycoplasma genitalium (M. genitalium)*, and *Mycoplasma hominis (M. hominis)*. Specimens were collected for HPV genotyping based on clinical diagnoses, including external genital swabs, urethral swabs, urine, and semen. Cases were classified into six categories based on the final clinical diagnosis (Figure 1). Follow-up indicators included HPV complete clearance, isolated persistence, incident-only genotype, and superinfection. This retrospective study was approved by the Ethics Committee of Wenzhou People’s Hospital and Wenzhou Maternal and Child Health Hospital. Inclusion criteria: (1) Male outpatients presenting for their first visit with clinical suspicion of genital warts, genital tract infections, or other conditions requiring pathogen detection. (2) Complete clinical medical records, sampling, laboratory test results and follow-up data. Exclusion criteria: (1) Patients with severe dysfunction of the heart, liver, kidney and other vital organs, or malignant tumors. (2) Patients with autoimmune diseases or long-term use of systemic glucocorticoids and immunosuppressants. (3) Patients who took systemic antiviral or antibacterial drugs within 30 days prior to baseline sampling.

### 2.2. Methods

#### 2.2.1. Specimen Collection

Specimens were collected by trained clinicians according to each patient’s clinical presentation. For patients with urethral symptoms or suspected urethritis, urethral discharge was obtained using a sterile swab inserted 1–2 cm into the urethra and rotated gently. For patients without obvious urethral discharge, first-void urine (10–20 mL) was collected in a sterile container. For patients attending the reproductive medicine center with fertility concerns, semen samples were collected by masturbation after 2–7 days of abstinence. For patients with genital lesions or suspected external genital infection, genital exudate was collected by swabbing the lesion surface. All specimens were immediately placed in dedicated viral transport medium, stored at 2–8 °C, and transported to the laboratory within 2 h for nucleic acid extraction and HPV genotyping.

#### 2.2.2. Gene Amplification and Genotyping

Human HPV nucleic acid detection and genotyping reagents (Haishi Gene Technology Co., Ltd., Ningbo, China) covered 17 high-risk genotypes (16, 18, 26, 31, 33, 35, 39, 45, 51, 52, 56, 58, 59, 66, 68, 73, 82) and 8 low-risk genotypes (6, 11, 40, 42, 43, 44, 81, 83). The following instruments were used: GeneRotex 96 fully automatic nucleic acid extraction instrument (Tianlong Technology Co., Ltd., Suzhou, China), A200 gene amplification instrument (Langji Scientific Instrument Co., Ltd., Hangzhou, China), and 3500xL Dx genetic analyzer (Applied Biosystems, Foster City, CA, USA).

Multiplex PCR was performed under the following conditions: 42 °C for 5 min and initial denaturation at 94 °C for 8 min, followed by 35 cycles of denaturation at 94 °C for 30 s, annealing at 60 °C for 30 s, and extension at 70 °C for 1 min, with a final extension at 70 °C for 1 min. After amplification, 1 µL of PCR product and 9 µL of Hi-Di formamide (containing 2.5% SIZE-500) were added to each well, mixed, and briefly centrifuged. The mixture was denatured at 86 °C for 3 min and cooled at 4 °C for 3 min. Capillary electrophoresis was performed for fragment separation, and genotypes were determined using GeneMapper 5.0 software. Dual quality control was applied for both multiplex PCR and capillary electrophoresis. A β-globin signal below 150 indicated insufficient exfoliated cells, requiring specimen recollection, a pcDNA signal below 150 indicated failed DNA extraction or PCR amplification, requiring repeated testing.

### 2.3. Statistical Analysis

Statistical analysis was performed using SPSS 26.0. Categorical variables were presented as counts (*n*) and proportions (%). Between-group comparisons were conducted using the chi-square test. A two-sided *p* value < 0.05 was considered statistically significant. The 95% confidence intervals (CIs) for proportions were derived using the exact (Clopper-Pearson) method in SPSS.

## 3. Results

### 3.1. HPV Positivity Rates Across Subgroups

The study enrolled 1934 participants, among whom 757 (39.14%) tested HPV-positive. As shown in Table 1, no significant difference in HPV positivity was observed among age groups (*p* > 0.05), although a trend toward higher rates was noted among younger adults. The 21–29 age group had the highest positivity rate of 43.22% (95% CI: 39.10–47.30%), followed by the 30–39 age group (37.69%, 95% CI: 34.20–41.30%). Significant differences in HPV positivity were found among specimen type groups and clinical diagnosis groups (both *p* < 0.001). The highest positivity rate was observed in external genital specimens (44.51%, 95% CI: 41.76–47.29%), whereas semen specimens had the lowest rate (19.91%, 95% CI: 14.74–25.88%). Among clinical diagnosis groups, patients with mucocutaneous lesions had the highest HPV positivity rate (58.07%, 95% CI: 53.89–62.18%), and participants presenting for male reproductive-health-related consultations had the lowest HPV positivity rate (23.90%, 95% CI: 19.42–28.89%).

### 3.2. Concurrent Sexually Transmitted Infections

As shown in Table 2, the prevalence of concurrent STIs in this cohort was 36.04%. Although STI prevalence was 37.65% among HPV-positive men, higher than the 35.00% among HPV-negative men, no significant difference was observed between the two groups (*p* > 0.05). Individual statistical analyses of each pathogen revealed no statistically significant differences in the positivity rates of *Neisseria gonorrhoeae*, *Treponema pallidum*, and all types of non-gonococcal pathogens between HPV-positive and HPV-negative men (*p* > 0.05). Only one case of human immunodeficiency virus infection was identified in the cohort, and statistical comparison was not performed.

### 3.3. Distribution Characteristics of HPV Genotypes

Seventeen high-risk HPV genotypes were detected among 757 HPV-positive male participants. HPV52 was the most frequently detected high-risk genotype, with a positivity rate of 13.61% (95% CI: 11.12–16.43%), followed by HPV51 (9.91%, 95% CI: 7.86–12.29%), HPV16 (8.59%, 95% CI: 6.56–11.04%), HPV39 (7.40%, 95% CI: 5.60–9.61%), and HPV58 (6.87%, 95% CI: 5.16–8.89%). HPV73 showed the lowest positivity rate of 0.13% (95% CI: 0.00–0.74%). In addition, eight low-risk HPV genotypes were identified. HPV6 was the predominant low-risk genotype, with a positivity rate of 40.16% (95% CI: 36.69–43.66%). Other common low-risk genotypes were HPV11 (9.38%, 95% CI: 7.46–11.62%), HPV42 (9.25%, 95% CI: 7.34–11.50%), HPV43 (7.79%, 95% CI: 6.00–9.92%), and HPV81 (6.87%, 95% CI: 5.16–8.89%). HPV40 had the lowest positivity rate among low-risk genotypes at 0.26% (95% CI: 0.03–0.93%). The positivity rates and 95% confidence intervals for all HPV genotypes are presented in Table 3.

### 3.4. HPV Positivity Rates Across Different Clinical Diseases

As shown in Figure 2, HPV positivity rates varied substantially across different clinical conditions among male outpatients. Patients with mucocutaneous lesions generally had relatively high HPV positivity rates. Within this cohort, patients with warts had the highest HPV positivity rate (63.17%), followed by skin ulcers (53.54%). Infectious diseases mainly included urinary tract infections and syphilis, with HPV positivity rates of 50.00% and 32.23%, respectively. Among inflammatory diseases, patients with scrotal eczema had the highest HPV positivity rate (39.30%), followed by cystitis (35.66%). For disorders related to male reproductive dysfunction, the HPV positivity rate was 39.39% for male infertility and 39.13% for premature ejaculation. Among proliferative and neoplastic-like lesions, patients with scrotal abscess had the highest HPV positivity rate (28.95%), followed by penile masses (20.00%).

### 3.5. Characteristics of HPV Infection Patterns

#### 3.5.1. Age Stratification of HPV Infection

The age-stratified distribution of HPV infection patterns among HPV-positive males is presented in Table 4. According to the number of infecting genotypes, single-genotype infection was the predominant pattern, accounting for 73.71% (95% CI: 70.58–76.85%), followed by double HPV genotypes (19.02%, 95% CI: 16.23–21.82%). Chi-square tests showed that only the proportion of triple-genotype co-infection differed significantly across age groups (*p* < 0.001). When infections were categorized according to the oncogenic risk of HPV genotypes, high-risk-only infection accounted for 48.08% (95% CI: 44.53–51.64%), low-risk-only infection for 36.99% (95% CI: 33.55–40.43%), and mixed high- and low-risk co-infection for 14.93% (95% CI: 12.39–17.47%). Chi-square testing revealed a significant age-related difference in the proportion of exclusive high-risk HPV infection (*p* < 0.05), whereas the distributions of low-risk-only infection and mixed-type infection were consistent across age groups (all *p* > 0.05).

#### 3.5.2. HPV Co-Infection and Disease Distribution

Figure 3 illustrates pairwise co-occurrence features of HPV genotypes and their distribution across different clinical diseases. As shown in Figure 3A, the colour intensity of each square indicated the frequency of co-detection for two HPV genotypes. Among low-risk genotypes, HPV6 exhibited the most extensive co-infection relationships, with the highest co-occurrence frequencies observed with HPV11 and HPV42. For high-risk genotypes, pairwise co-infection between HPV52 and HPV51 or HPV58 was most prominent. In contrast, high-risk genotypes including HPV16, HPV18, HPV26, and HPV31 seldom formed co-infection pairs. In terms of cross-risk-group co-occurrence, HPV6 was co-detected with the locally predominant high-risk genotypes HPV52 and HPV51, whereas cross-risk combinations among other genotypes were relatively rare. Figure 3B was a circular stacked plot that showed the positive-case frequency distribution of 25 HPV genotypes across six categories of urogenital disorders. Low-risk HPV6 yielded the largest number of positive cases and was predominantly concentrated in mucocutaneous lesions. The locally prevalent high-risk genotypes HPV52, HPV51, and HPV58 were mostly distributed among inflammatory diseases and male reproductive-related disorders. HPV16 and HPV18 were infrequently detected and showed no obvious clustering within any disease category.

### 3.6. Analysis of Six-Month HPV Follow-Up Outcomes in Males

Among 757 male outpatients with positive baseline HPV results, 403 completed the six-month follow-up, yielding an overall follow-up completion rate of 53.24%. Baseline demographic and virological characteristics, including age distribution, infection multiplicity, and baseline HPV risk category, were compared between participants who completed follow-up and those lost to follow-up. All virological-related baseline variables were well-balanced between the two groups (all *p* > 0.05), supporting the reliability of virological follow-up outcomes (Table 5). Differences in clinical presentation existed between groups; patients with mild symptoms tended to discontinue follow-up, whereas individuals with obvious clinical manifestations were more likely to return for re-examination.

As shown in Table 6, stratified analysis by age revealed no significant between-group differences in follow-up completion rate, isolated persistence rate, or incident-only genotype rate (all *p* > 0.05). By contrast, significant between-group differences were observed for complete clearance rate and superinfection rate (both *p* < 0.05). Participants aged ≤29 years had lower complete clearance rates and substantially higher superinfection rates; the superinfection rate reached 27.27% in the ≤20-year-old subgroup. When stratified by baseline HPV infection type, follow-up completion rates were comparable across the high-risk, low-risk, and mixed high- and low-risk groups (all *p* > 0.05). No between-group difference was noted for the incident-only genotype rate (*p* > 0.05). In contrast, highly significant intergroup differences were found in complete clearance rate, isolated persistence rate, and superinfection rate (all *p* < 0.001).

## 4. Discussion

Current HPV research and prevention strategies focus heavily on female cervical diseases, causing prominent gender disparity in relevant evidence and clinical practice. The epidemiological features, genotypic profiles, and clinical significance of male HPV infection remain undercharacterized. As key asymptomatic carriers and transmission vectors, males often harbor latent or multiple-genotype HPV infections, driving ongoing partner-to-partner transmission and hindering population-level cervical cancer prevention [12]. While prior studies have identified behavioral factors, HIV status, circumcision, and vaccination as male HPV risk factors, these findings have not been translated into standardized male screening guidelines. A critical gap exists in the necessity and operational protocols for routine male HPV screening, especially in Wenzhou, where local data on male HPV epidemiology and genotype–lesion associations are scarce.

To address this gap and facilitate gender-balanced HPV prevention, this study analyzed real-world outpatient data to characterize local male HPV epidemiology, genotypic heterogeneity, co-infection patterns, and genotype–disease correlations. Six-month follow-up outcomes stratified by age and baseline infection status were further evaluated to inform targeted regional screening and individualized management strategies.

### 4.1. Population Epidemiological Distribution Characteristics

A total of 1934 valid male clinical specimens were analyzed, yielding a local outpatient HPV positivity rate of 39.14%, which is markedly higher than the 15% pooled prevalence (95% CI: 11–21%) in East and Southeast Asian males [13]. Domestic studies have reported lower positivity rates in northern, central, and southwestern Chinese regions, as well as coastal metropolises including Shanghai and Guangzhou [14,15,16,17,18,19,20]. Such regional discrepancies are mainly attributed to uneven medical resources, variable public health awareness, and regional differences in HPV prevention implementation. Developed coastal cities have mature male HPV health education and vaccination systems, while Wenzhou’s active population mobility accelerates viral transmission, and low local screening and vaccination coverage elevates population infection burden. These findings highlight the necessity of region-specific screening, health education, and prevention strategies for local males.

Similarly, large-scale local studies of Wenzhou female outpatients have reported an overall HPV positivity rate of 17–20%, with high-risk genotypes HPV16, 52, and 58 predominating [21,22]. Our male outpatient cohort showed this markedly higher positivity rate, dominated by low-risk HPV6 and HPV11. Such gender disparities in infection prevalence and genotype spectrum stem from differences in sampling sites, clinical populations, and genital microenvironments. The abundant mucosal folds and moist, enclosed microenvironment of the male external genitalia favor persistent colonization and latent infection by low-risk HPV. Men often carry HPV asymptomatically, and the lack of routine male screening further elevates detection rates. Marked gender gaps exist in HPV vaccination across China. Women demonstrate greater health awareness and higher vaccine uptake, whereas low vaccination coverage among males results in inadequate herd protection. Notably, China’s National Medical Products Administration approved the quadrivalent HPV vaccine for males aged 9–26 years in January 2025 and the nonavalent HPV vaccine for males aged 16–26 years in April 2025, marking a major milestone for male HPV prevention in China. Our findings highlight the public health value of HPV vaccination in men. Gender-balanced prevention strategies can reduce the burden of HPV-associated diseases in males, interrupt sexual transmission, and lower the risk of cross-infection between sexual partners.

Although overall HPV positivity showed no significant age-group difference, distinct age-dependent infection trends were observed. The 21–29 age group had the highest positivity rate (43.22%), followed by the 30–39 and 40–49 age groups, forming a unimodal peak among young and middle-aged males. This differs from the bimodal age distribution in northern Chinese cohorts. Young and middle-aged males constitute the core high-risk group, attributed to higher sexual activity and insufficient awareness of HPV prevention. Unlike the bimodal prevalence pattern in Chinese females, this male-specific unimodal peak reflects significant gender divergence in HPV transmission and natural history. This unique regional pattern may stem from outpatient sampling characteristics and local behavioral features, demonstrating prominent regional heterogeneity in male HPV age distribution across China.

In addition, this study found that the prevalence of concurrent STIs was higher in HPV-positive than HPV-negative patients, but the between-group difference was not statistically significant (*p* > 0.05). This is likely attributable to the cohort comprising predominantly general male outpatients with low STI-related high-risk exposure, which limited our ability to detect a significant association between HPV and co-infection with other genital pathogens.

### 4.2. Genotype Epidemiological Heterogeneity

Based on overall epidemiological data, we further analyzed genotypic distribution among HPV-positive males and identified notable regional genotype heterogeneity. The dominant high-risk genotype was HPV52 (13.61%), followed by HPV51, HPV16, HPV39, and HPV58, while rare high-risk genotypes (HPV73, HPV26) showed extremely low prevalence. National dominant HPV genotypes are primarily derived from female populations, with HPV16/18 as the predominant high-risk genotypes [23,24]. In contrast, local male high-risk infections were dominated by non-16/18 genotypes, indicating region-specific epidemic characteristics and limiting the applicability of female-oriented national prevention strategies to local males. For low-risk genotypes, HPV6 was overwhelmingly predominant (40.16%), far exceeding other low-risk genotypes and largely accounting for local male HPV-related mucosal and cutaneous lesions. Although HPV genotype spectra vary considerably across study populations and clinical settings, it is noteworthy that the prevalence of a specific genotype in infected individuals does not equate to its oncogenic potential. In our symptomatic male cohort, low-risk HPV6 showed the highest detection rate, while high-risk HPV52/51 exhibited moderate prevalence; nevertheless, these locally circulating high-risk genotypes retain strong malignant transformation capacity. Therefore, our six-month follow-up findings further indicate the need for long-term monitoring of non-16/18 high-risk HPV infections among local males. Overall, the local male HPV disease burden is mainly attributable to low-risk genotypes and non-16/18 high-risk genotypes, rather than classical oncogenic HPV16/18, providing genotype-specific evidence for regional prevention and vaccination optimization.

### 4.3. Co-Infection Patterns and Genotype–Disease Correlations

This study further investigated infection patterns and corresponding clinical manifestations among HPV-positive males. Single-genotype infection predominated (73.71%), while multiple-genotype co-infection accounted for a relatively small proportion. This distribution pattern is consistent with previous domestic and international epidemiological findings on male HPV infection [13,25]. To comprehensively characterize local co-infection features, we analyzed prevalent genotype combinations and evaluated the association between individual genotypes and clinical lesions. Low-risk HPV6 constituted the most common component of mixed infections and frequently co-occurred with HPV11, HPV42, and HPV43. The locally dominant high-risk genotype HPV52 commonly co-existed with HPV51 and HPV58, whereas HPV16/18 were rarely detected in co-infection cases. Most cross-risk co-infections involved HPV6 combined with locally circulating high-risk genotypes. Distinct clinical correlations were observed among different genotypes. Low-risk genotypes were predominantly detected in patients with genital warts and mucosal ulcers. Local high-risk genotypes showed moderate correlations with urinary tract inflammation and reproductive dysfunction. In contrast, few HPV genotypes were identified in proliferative or neoplastic lesions, indicating a weak pathogenic association between HPV infection and male neoplastic diseases in this population.

Age-stratified analysis showed that participants aged ≤20 years had the highest triple-genotype infection rate, with co-infection prevalence declining with age. Younger males were prone to low-risk and multiple-genotype infections, while middle-aged and elderly males tended to develop persistent high-risk single-genotype infection, consistent with previous Chinese male cohort studies [26,27]. Adolescent viral exposure causes transient multiple-genotype co-infection, whereas adult immune surveillance eliminates most low-risk viruses, ultimately leading to persistent single high-risk genotype infection, a core feature of male HPV natural history.

Beyond individual age effects, geography also shapes male HPV co-infection profiles with significant interregional heterogeneity. In the present Wenzhou cohort, multiple-genotype co-infection accounted for 26.29% of HPV-positive cases, which was lower than the rates reported in other southern Chinese cities, including Shantou (37.57%), Qingyuan (35.72%), and Huizhou (35.71%) [7,25,28]. Across these southern regions, co-infections were consistently dominated by low-risk pairs (HPV6+11/42/43) or high-risk pairs (HPV52+51/58), whereas HPV16/18 were rarely involved. The relatively lower co-infection rate in Wenzhou may reflect differences in study population composition, specimen collection strategies, and local sexual-behavioral characteristics. Findings from Southeast Asian cohorts and the international HIM trial further validate such geographic divergence in co-infection patterns [29,30].

### 4.4. Follow-Up and Management Strategies

A six-month prospective follow-up was conducted in 757 HPV-positive males, with valid prognostic data obtained from 403 participants. Stratified chi-square analysis showed comparable follow-up completion rates across age groups (*p* > 0.05), indicating balanced baseline conditions and minimal selection bias. Age and baseline multiple-genotype co-infection were independent determinants of short-term HPV natural history, including spontaneous clearance, persistent infection, novel genotype acquisition, and superinfection. Distinct age-related prognostic differences were observed: the 30–49-year age group had the highest spontaneous clearance rate due to mature immune function; males aged ≤20 years had the lowest clearance efficiency and highest superinfection risk, related to frequent sexual exposure and immature immune regulation; adults aged ≥60 years exhibited impaired viral clearance without elevated multiple-genotype infection risk, with single-genotype persistent infection as the main outcome. Consistent single-genotype persistence and novel genotype acquisition rates across age groups confirm that single-genotype persistent infection is driven by viral immune escape rather than age. Baseline infection status significantly affected prognosis: single-genotype infections had higher clearance potential, while multiple-genotype co-infections had only 11.29% full clearance and 20.97% superinfection rates. Concurrent carriage of multiple genotypes disrupts genital mucosal immune homeostasis, impedes viral elimination, increases novel genotype susceptibility, and forms a vicious cycle of persistent mixed infection.

These findings support stratified, individualized management strategies for male HPV infection. Young males and patients with baseline multiple-genotype co-infection are high-risk groups for viral persistence and superinfection, requiring intensified surveillance. Targeted adolescent health education is essential to reduce diverse HPV genotype exposure. Shortened follow-up intervals are recommended for multiple-genotype co-infection cases due to poor spontaneous clearance and high superinfection risk, while males aged 30–49 years with robust viral clearance can be managed with routine follow-up protocols.

## 5. Limitations

This study has several limitations. First, although sexual behavioural factors and partner infection status are established HPV risk factors, they were not included in our analytical model. These unmeasured confounders may bias the observed association between HPV infection and male urogenital lesions. Second, the six-month follow-up period only enabled evaluation of short-term viral prognosis, failing to reflect long-term viral clearance dynamics and lesion progression. However, conducting long-term follow-up in routine clinical settings is challenging. Most infected individuals have insufficient awareness of HPV-related risks and low willingness for re-examination. In addition, our participants were outpatients, making sustained long-term contact difficult to maintain. Third, the follow-up rate of this study was only 53.24%. This partly reflects inadequate public attention to male HPV surveillance. Routine male HPV screening is not implemented nationwide in China, standardised follow-up protocols are lacking, and the absence of systematic patient reminder mechanisms further contributed to loss to follow-up. Despite these limitations, this study provides valuable local real-world epidemiological evidence, supporting short-term risk stratification and individualised follow-up design for HPV-positive males in this region.

## 6. Conclusions

In summary, this study systematically characterized male HPV infection in Wenzhou across multiple dimensions, including population-level epidemiological patterns, genotypic heterogeneity, co-infection characteristics, and genotype–disease associations, supplemented by six-month follow-up outcomes stratified by age and baseline infection status. Against the backdrop of expanded eligibility for HPV vaccination in China, this study supports the implementation of a three-pronged strategy combining screening, vaccination and health education to correct the gender bias that prioritizes females in HPV prevention and improve men’s willingness to undergo screening and their overall prevention awareness. The findings can inform the development of a gender-equitable, comprehensive HPV prevention system.

## Figures and Tables

**Figure 1:**
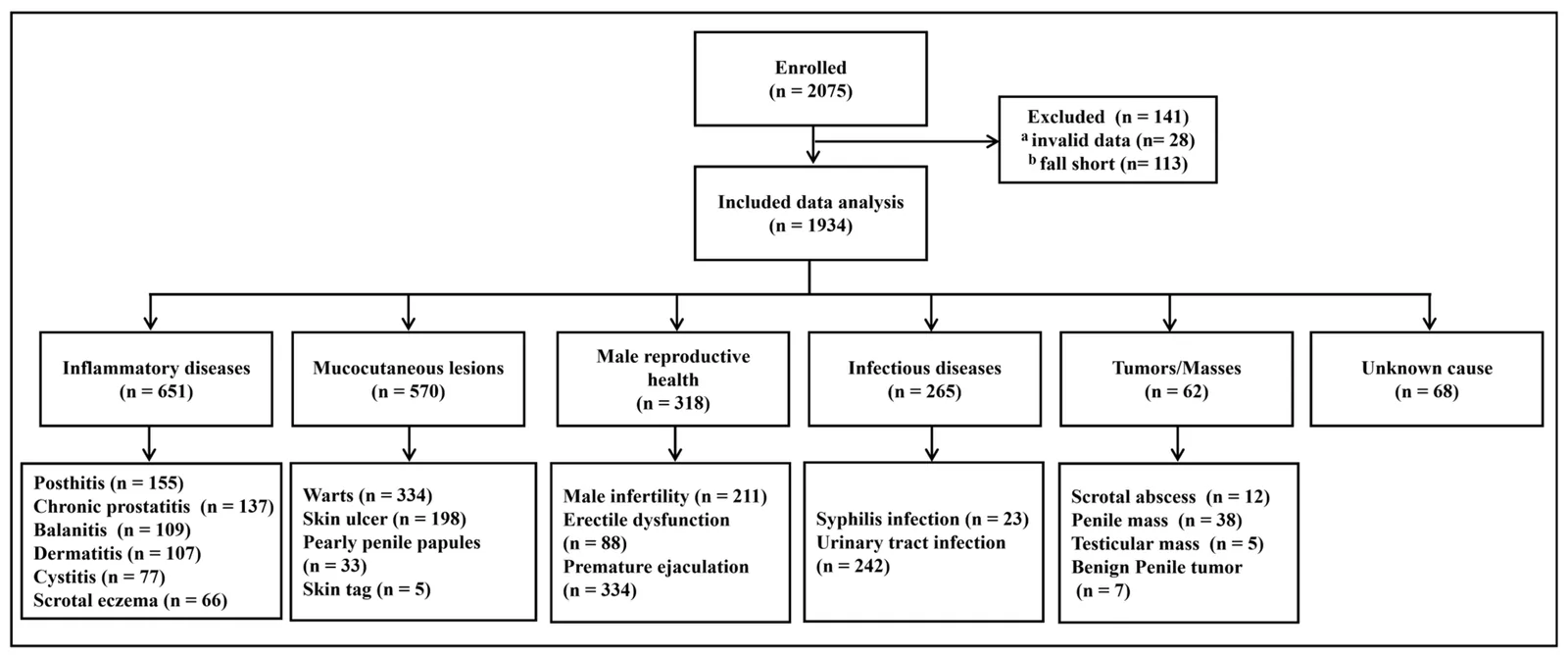
Flowchart of participant screening and grouping. ^a^ Samples with insufficient DNA. ^b^ Incomplete clinical data.

**Figure 2:**
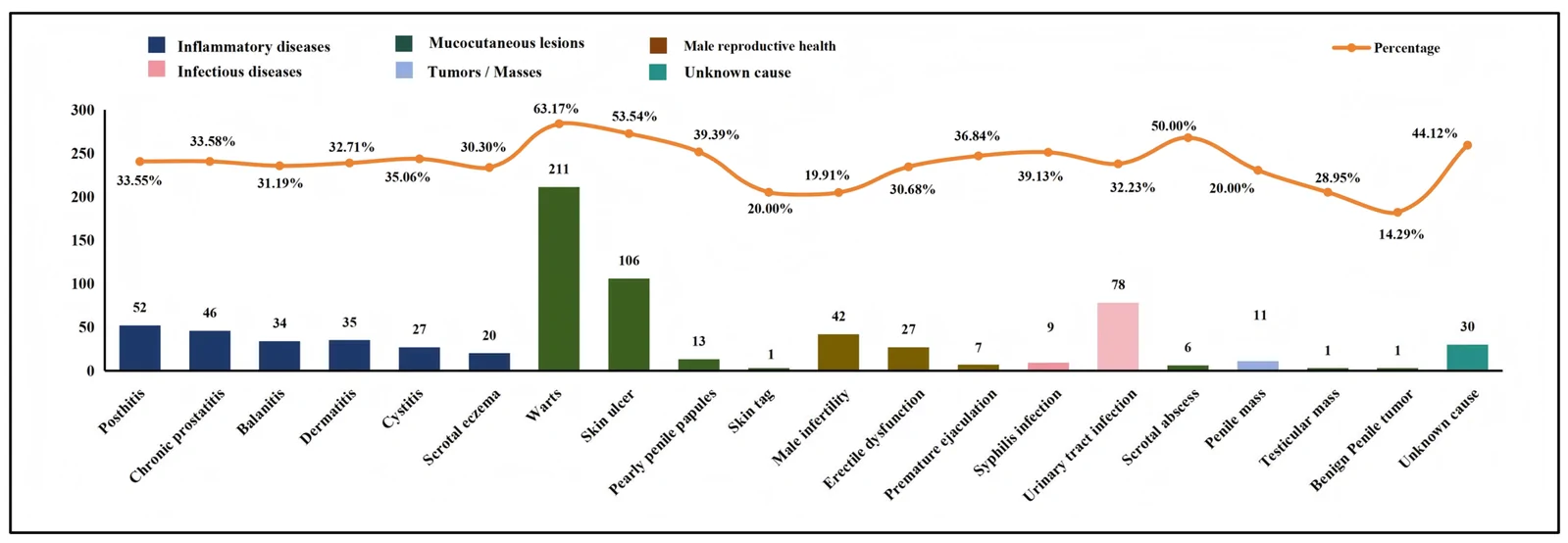
HPV positivity rate among males with different clinical diagnoses.

**Figure 3:**
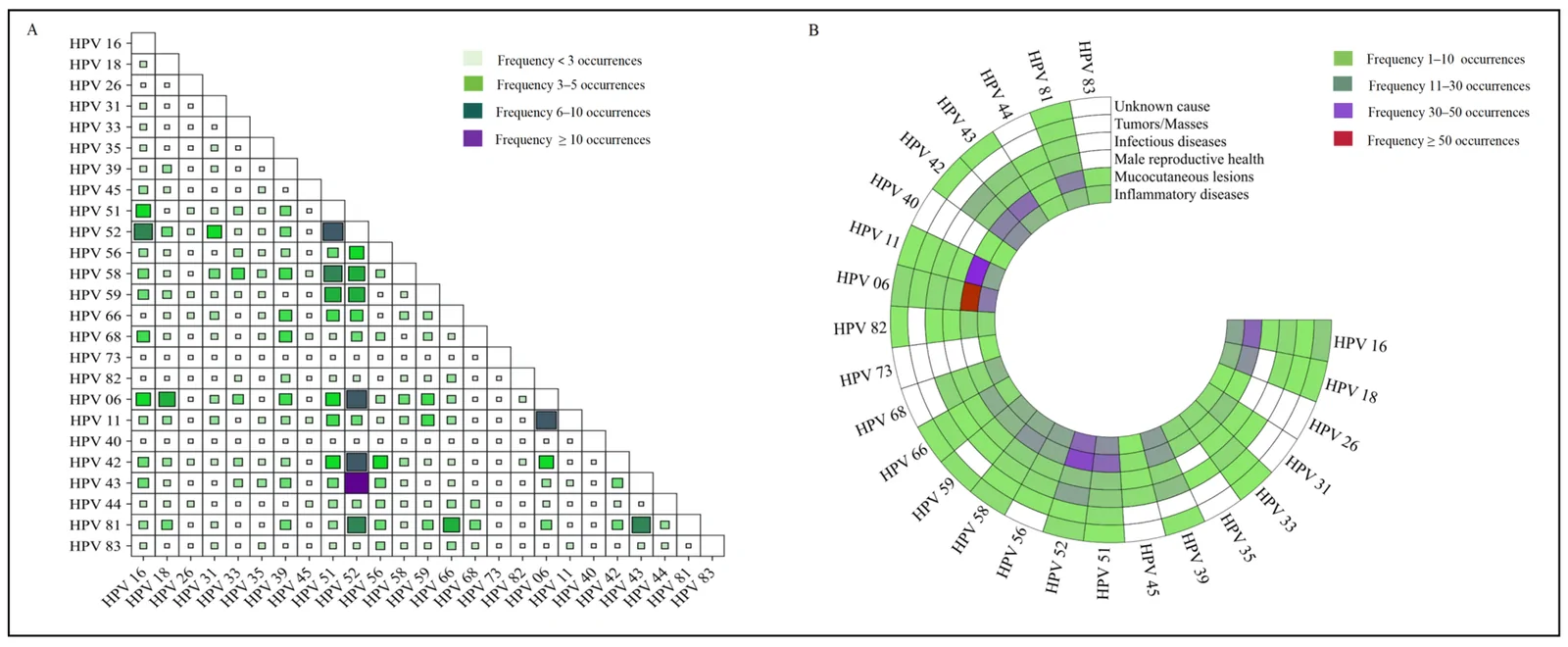
Pairwise co-occurrence of HPV genotypes and their distribution among urogenital diseases. (**A**) Lower-triangular heatmap, where both color intensity and square size represent co-detection frequency of HPV genotype pairs. (**B**) Circular stacked plot presenting positive-case frequencies of 25 HPV genotypes across six urogenital disease categories.

**Table 1:** HPV positivity rates by participant characteristics.

Characteristic	Total	HPV-Positive	95% CI (%)	*χ* ^2^	*p* Value
	(*n* = 1934)	(*n* = 757)			
Age				10.251	0.068
≤20 years	54 (2.79)	20 (2.64)	37.04 (24.29–51.20)		
21–29 years	583 (30.14)	252 (33.29)	43.22 (39.10–47.30)		
30–39 years	743 (38.42)	280 (36.99)	37.69 (34.20–41.30)		
40–49 years	342 (17.68)	115 (15.19)	33.63 (29.20–39.40)		
50–59 years	155 (8.01)	65 (8.59)	41.94 (34.10–50.10)		
≥60 years	57 (2.95)	25 (3.30)	43.85 (30.50–57.60)		
Marital status				6.064	0.014
Unmarried	679 (35.11)	291 (38.44)	42.86 (39.11–46.66)		
Married or cohabiting	1255 (64.89)	466 (61.56)	37.13 (34.43–39.89)		
Specimen type				55.105	<0.001
External genitalia	1267 (65.51)	564 (74.50)	44.51 (41.76–47.29)		
Urethra	137 (7.08)	46 (6.08)	33.58 (25.76–42.19)		
Urine	319 (16.49)	105 (13.87)	32.92 (27.82–38.39)		
Semen	211 (10.91)	42 (5.55)	19.91 (14.74–25.88)		
Clinical diagnosis				134.454	<0.001
Inflammatory diseases	651 (33.66)	214 (28.27)	32.82 (29.29–36.50)		
Mucocutaneous lesions	570 (29.47)	331 (43.73)	58.07 (53.89–62.18)		
Male reproductive health	318 (16.44)	76 (10.04)	23.90 (19.42–28.89)		
Infectious diseases	265 (13.70)	87 (11.49)	32.83 (27.29–38.84)		
Tumors/Masses	62 (3.21)	19 (2.51)	31.15 (20.28–43.97)		
Unknown cause	68 (3.52)	30 (3.96)	44.12 (31.73–57.04)		

**Table 2:** Concurrent sexually transmitted infections in 1934 men stratified by HPV status.

Concurrent STIs	Total	HPV-Positive	HPV-Negative	*χ* ^2^	*p* Value
	(*n* = 1934)	(*n* = 757)	(*n* = 1177)		
HIV	1 (0.05)	1 (0.13)	0 (0.00)	—	—
*T. pallidum*	23 (1.19)	8 (1.06)	15 (1.27)	0.367	0.545
*N. gonorrhoeae*	76 (3.93)	28 (3.70)	48 (4.08)	0.578	0.447
*U. urealyticum*	483 (24.97)	205 (27.08)	278 (23.62)	2.946	0.086
*C. trachomatis*	90 (4.65)	37 (4.89)	53 (4.50)	0.154	0.695
*M. genitalium*	23 (1.19)	12 (1.59)	11 (0.93)	1.660	0.198
*M. hominis*	61 (3.15)	30 (3.96)	31 (2.63)	2.665	0.103
Total	697 (36.04)	285 (37.65)	412 (35.00)	1.398	0.237

Statistical analysis was not performed for HIV due to the small number of positive cases.

**Table 3:** Genotype detection results among HPV-positive male patients.

	Genotype	HPV-Positive (*n* = 757)	95% CI	CI Width (%)
High-Risk	HPV16	65	8.59 (6.56–11.04)	4.48
	HPV18	40	5.28 (3.76–7.22)	3.46
	HPV26	2	0.26 (0.03–0.93)	0.9
	HPV31	16	2.11 (1.22–3.43)	2.21
	HPV33	18	2.38 (1.42–3.81)	2.39
	HPV35	12	1.59 (0.82–2.81)	1.99
	HPV39	56	7.40 (5.60–9.61)	4.01
	HPV45	10	1.32 (0.62–2.52)	1.9
	HPV51	75	9.91 (7.86–12.29)	4.43
	HPV52	103	13.61 (11.12–16.43)	5.31
	HPV56	48	6.34 (5.00–7.99)	2.99
	HPV58	52	6.87 (5.16–8.89)	3.73
	HPV59	36	4.76 (3.32–6.46)	3.14
	HPV66	29	3.83 (2.56–5.36)	2.8
	HPV68	28	3.70 (2.46–5.23)	2.77
	HPV73	1	0.13 (0.00–0.74)	0.74
	HPV82	14	1.85 (0.98–3.20)	2.22
Low-Risk	HPV6	304	40.16 (36.69–43.66)	6.97
	HPV11	71	9.38 (7.46–11.62)	4.16
	HPV40	2	0.26 (0.03–0.93)	0.9
	HPV42	70	9.25 (7.34–11.50)	4.16
	HPV43	59	7.79 (6.00–9.92)	3.92
	HPV44	14	1.85 (0.98–3.20)	2.22
	HPV81	52	6.87 (5.16–8.89)	3.73
	HPV83	9	1.19 (0.52–2.30)	1.78

**Table 4:** Age distribution of infection patterns in HPV-positive males.

	Total (*n* = 757)	95% CI (%)	≤20 Years	21~29 Years	30~39 Years	40~49 Years	50~59 Years	≥60 Years	*χ* ^2^	*p* Value
			*n* = 20	*n* = 252	*n* = 280	*n* = 115	*n* = 65	*n* = 25		
Infection pattern										
Single HPV genotype	558 (73.71)	73.71 (70.58–76.85)	13 (65.00)	180 (71.43)	214 (76.43)	90 (78.26)	42 (64.62)	19 (76.00)	6.599	0.525
Double HPV genotypes	144 (19.02)	19.02 (16.23–21.82)	2 (10.00)	46 (18.25)	52 (18.57)	22 (19.13)	18 (27.69)	4 (16.00)	4.511	0.478
Triple HPV genotypes	39 (5.15)	5.15 (3.58–6.73)	4 (20.00)	19 (7.54)	7 (2.50)	2 (1.74)	5 (7.69)	2 (8.00)	20.008	0.001
Four or more HPV genotypes	16 (2.11)	2.11 (1.09–3.14)	1 (5.00)	7 (7.54)	7 (2.50)	1 (0.87)	0 (0.00)	0 (0.00)	4.348	0.500
Infection types										
High-Risk	364 (48.08)	48.08 (44.53–51.64)	6 (30.00)	103 (40.87)	144 (51.43)	63 (54.78)	36 (55.38)	12 (48.00)	12.579	0.028
Low-Risk	280 (36.99)	36.99 (33.55–40.43)	11 (55.00)	100 (39.68)	108 (38.57)	36 (31.30)	16 (24.62)	9 (36.00)	9.744	0.083
Mixed High-Risk and Low-Risk	113 (14.93)	14.93 (12.39–17.47)	3 (15.00)	49 (19.44)	28 (10.00)	16 (13.91)	13 (20.00)	4 (16.00)	10.835	0.055

**Table 5:** Baseline comparison of follow-up status.

Variables	*n* = 757	Completed Follow-Up (*n* = 403)	Lost to Follow-Up (*n* = 354)	*χ* ^2^	*p* Value
Age				6.490	0.261
≤20 years	20 (2.64)	11 (2.73)	9 (2.54)		
21–29 years	252 (33.29)	122 (30.27)	130 (36.72)		
30–39 years	280 (36.99)	149 (36.97)	131 (37.01)		
40–49 years	115 (15.19)	64 (15.88)	51 (14.41)		
50–59 years	65 (8.59)	42 (10.42)	23 (6.50)		
≥60 years	25 (3.30)	15 (3.72)	10 (2.82)		
Infection multiplicity				0.668	0.414
Single HPV genotype	558 (73.71)	302 (74.94)	256 (72.32)		
Multiple HPV genotypes	199 (26.29)	101 (25.06)	98 (27.68)		
Baseline HPV risk type				2.135	0.344
High-Risk	363 (47.95)	200 (49.63)	163 (46.05)		
Low-Risk	283 (37.38)	141 (34.99)	142 (40.11)		
Mixed High-Risk and Low-Risk	111 (14.66)	62 (15.38)	49 (13.84)		
Clinical diagnosis				96.147	<0.001
Inflammatory diseases	214 (28.27)	87 (21.59)	127 (35.88)		
Mucocutaneous lesions	331 (43.73)	157 (38.96)	174 (49.15)		
Male reproductive health	76 (10.04)	65 (16.13)	11 (3.11)		
Infectious diseases	87 (11.49)	71 (17.62)	16 (4.52)		
Tumors/Masses	19 (2.51)	16 (3.97)	3 (0.85)		
Unknown cause	30 (3.96)	7 (1.74)	23 (6.50)		

**Table 6:** Comparison of six-month follow-up outcomes in HPV-positive males.

Stratification Dimension	Group	HPV-Positive (*n* = 757)	Completed Follow-Up Cases (*n* = 403)	Complete Clearance Rate (*n* = 212)	Isolated Persistent Rate (*n* = 113)	Incident-Only Genotype Rate (*n* = 54)	Superinfection Rate (*n* = 24)
Age group	≤20 years	20	11 (55.00)	1 (9.09)	4 (36.36)	3 (27.27)	3 (27.27)
	21–29 years	252	122 (48.41)	60 (49.18)	34 (27.87)	18 (14.75)	10 (8.20)
	30–39 years	280	149 (53.21)	81 (54.36)	45 (30.20)	17 (11.41)	6 (4.03)
	40–49 years	115	64 (55.65)	40 (62.50)	14 (21.88)	8 (12.50)	2 (3.13)
	50–59 years	65	42 (64.62)	24 (57.14)	11 (26.19)	5 (11.90)	2 (4.76)
	≥60 years	25	15 (60.00)	6 (40.00)	5 (33.33)	3 (20.00)	1 (6.67)
	*χ* ^2^		6.490	12.929	2.209	3.215	12.470
	*p* value		0.261	0.024	0.819	0.667	0.029
HPV genotype	High-Risk	364	201 (55.22)	126 (62.69)	44 (21.89)	25 (12.44)	6 (2.99)
	Low-Risk	280	140 (50.00)	79 (56.43)	32 (22.86)	24 (17.14)	5 (3.57)
	Mixed High-Risk and Low-Risk	113	62 (54.87)	7 (11.29)	37 (20.97)	5 (8.06)	13 (20.97)
	*χ* ^2^		1.874	51.461	36.387	3.372	29.536
	*p* value		0.392	<0.001	<0.001	0.185	<0.001

Complete clearance: all baseline HPV genotypes became negative at follow-up. Isolated persistent: baseline HPV genotype(s) remained positive without incident new genotypes. Incident-only genotype: all baseline genotypes cleared, only newly acquired HPV genotypes detected at follow-up. Superinfection: persistence of baseline HPV genotype(s) together with newly incident HPV genotypes.

## Data Availability

All relevant data generated and analyzed during this study are included within the manuscript.
